# Lifespan Extending and Oxidative Stress Resistance Properties of a Leaf Extracts from *Anacardium occidentale* L. in *Caenorhabditis elegans*

**DOI:** 10.1155/2019/9012396

**Published:** 2019-06-02

**Authors:** Chatrawee Duangjan, Panthakarn Rangsinth, Xiaojie Gu, Michael Wink, Tewin Tencomnao

**Affiliations:** ^1^Graduate Program in Clinical Biochemistry and Molecular Medicine, Department of Clinical Chemistry, Faculty of Allied Health Sciences, Chulalongkorn University, Bangkok 10330, Thailand; ^2^Institute of Pharmacy and Molecular Biotechnology, Heidelberg University, Im Neuenheimer Feld 364, 69120 Heidelberg, Germany; ^3^Age-Related Inflammation and Degeneration Research Unit, Department of Clinical Chemistry, Faculty of Allied Health Sciences, Chulalongkorn University, Bangkok 10330, Thailand; ^4^Department of Biotechnology, School of Environmental and Chemical Engineering, Dalian Jiaotong University, Dalian 116028, China

## Abstract

*Anacardium occidentale* (AO) contains a number of polyphenolic secondary metabolites with antioxidant activity. The objectives of this study were aimed at investigating the roles of AO leaf extracts in antioxidative stress and longevity, as well as their underlying mechanisms, in the *Caenorhabditis elegans* (*C. elegans*) model. AO extracts mediated the survival rate of nematodes under oxidative stress by attenuating intracellular reactive oxygen species (ROS) via the DAF-16/FoxO and SKN-1/Nrf-2 signaling pathways. AO extracts stimulated the expression of stress response genes including SOD-3 and GST-4. Moreover, AO extracts exhibited antiaging activities and enhanced longevity. We observed improved pharyngeal pumping function, attenuation of pigment accumulation (lipofuscin), and an increased lifespan of the worms. Collectively, our results demonstrated that AO extracts exerted both oxidative stress resistance and antiaging properties in the *C. elegans* model and may lead to new agents to benefit humans in the near future.

## 1. Introduction

Long life and healthy aging depend on many fold interactions among biological and environmental factors. Aging is an inevitably natural process accompanied by accumulation of damaged macromolecules such as nucleic acids, lipids, and proteins. Consequently, physiological characteristic changes such as increased oxidative stress and increased inflammation can negatively affect the quality of life [1]. Although the mechanisms of the aging process are not completely understood, increasing evidence suggests that aging is apparently associated with the bioactivity of reactive oxygen species (ROS). The protective effects of ROS are the strategy to delay aging and related degenerative diseases. Several lines of evidence previously reported that the reduction of ROS and low-grade inflammation can extend lifespan in a wide spectrum of model organisms [1, 2].

The free-living soil nematode *C. elegans* has become a valuable model for studying genetic and pharmacological influences of ROS on health and longevity. *C. elegans* has a rapid reproduction rate and a short lifespan and is easy to maintain [3]. Its genome has been completely sequenced, and various transgenic strains are available for experimental studies [4]. Importantly, *C. elegans* has conserved longevity and stress resistance genes that are homologous to human genes and thus can serve as a model for human aging processes [5]. Therefore, *C. elegans* has become a popular model organism to explore the potential antiaging and stress resistance properties of natural compounds.

The insulin/IGF-1 signaling (IIS) pathway is one of the most well-known pathways studied in *C. elegans*, which is involved in the regulation of nutrient level responses via the forkhead box O (FoxO) transcription factor and its downstream targets. The components of the IIS pathway are well conserved and are linked to longevity in *C. elegans* and humans as well [6]. In *C. elegans,* the FoxO transcription factor DAF-16 plays a role in metabolism, dauer formation, stress resistance, and lifespan modulation [7, 8]. In addition, the transcriptional target genes of DAF-16, including superoxide dismutase-3 (SOD-3), catalase-1 (CTL-1), and small heat shock protein-16.2 (HSP-16.2), are key factors that contribute to mediating oxidative stress and heat shock stress response [9–11]. The SKN-1/Nrf-2 signaling pathway, with the transcription factor SKN-1, is localized in the intestine. It is regulated and influenced by growth, nutrients, and metabolic signals in *C. elegans*. SKN-1 is also involved in acute stress response functions by regulating its downstream targets such as glutathione S-transferase 4 (GST-4), which is a phase II detoxification enzyme. Importantly, SKN-1 plays a central role in many regulatory pathways and interventions that extend lifespan in *C. elegans* [12]. A previous report suggests that both DAF-16 and SKN-1 promote stress resistance and, consequently, lifespan extension [13].

The cashew tree *Anacardium occidentale* L. (AO), which is known as mamuanghimmaphan in Thai, belongs to the family Anacardiaceae. It originates from Brazil but is presently cultivated in many tropical countries around the globe. A recent study reported that leaf extracts from *A. occidentale* are rich in beta-carotene, lutein, and polyphenols, which are known for high antioxidant activities [14].

As people want to live longer and healthier, healthy nutrition has increasingly received much attention in recent years. Several studies reported a positive correlation between antioxidants in foods, drinks, and longevity. A number of natural products have been reported to extend lifespan in *C. elegans*, such as a variety of antioxidant compounds [15, 16] including epigallocatechin gallate [17], quercetin [18], or anthocyanins [19]. Therefore, natural products from food supplements and medicinal plants with antioxidant properties could be promising candidates for fighting against various aging-related diseases and promoting longevity.

Leaf extracts from AO have been of great interests due to their pronounced antioxidant effects [20, 21]. However, the effect of AO leaf extracts with respect to age-related diseases and lifespan extension still needs to be elucidated. Therefore, the objectives of this study were aimed at investigating the modulatory roles and underlying mechanisms of AO leaf extracts on oxidative stress and lifespan extension in the *C. elegans* model. Our novel findings could initiate further applications of AO leaf extracts as natural products beneficial to humans in the future.

## 2. Materials and Methods

### 2.1. Chemicals and Reagents

Juglone (5-hydroxy-1,4-naphthoquinone) and 2,7-dichlorofluorescein diacetate (H2DCF-DA) were obtained from Sigma-Aldrich GmbH (Steinheim, Germany). Sodium azide was obtained from AppliChem GmbH (Darmstadt, Germany). EGCG (purity ≥ 95%) was purchased from Sigma-Aldrich (München, Germany). Dimethyl sulfoxide (DMSO), 2,2-azino-bis(3-ethylbenzothiazoline-6-sulfonic acid) diammonium salt (ABTS), 2,2-diphenyl-1-picrylhydrazyl (DPPH), Folin-Ciocalteu reagent, and quercetin were obtained from Sigma-Aldrich (MO, USA). Gallic acid was purchased from TCI America (OR, USA). L-Ascorbic acid was obtained from Calbiochem (CA, USA). Other reagents used for extraction were of analytical grade and were purchased from RCI Labscan (Bangkok, Thailand).

### 2.2. Plant Material and Extraction

#### 2.2.1. Plant Material

The leaves of *Anacardium occidentale* L. (AO) were collected from Jana district, Songkhla Province, in southern Thailand and were stored as a voucher specimen (no. BCU-015863) at the herbarium of Kasin Suvatabhandhu (Department of Botany, Faculty of Science, Chulalongkorn University, Thailand).

#### 2.2.2. Plant Extraction

The dried leaves of AO (40 g) were extracted sequentially with 400 mL hexane, dichloromethane, and methanol by Soxhlet for 36 h. The extracts were evaporated at 35-45°C after filtration using Whatman no. 1 filter paper. The residue was dissolved in dimethyl sulfoxide (DMSO) to a final concentration of 100 mg/mL as stock solution and stored at -20°C until needed for analysis.

### 2.3. Qualitative Phytochemical Screening

The hexane and methanol extracts were submitted to screening and phytochemical analysis by using GLC-MS and LC-MS (gas/liquid chromatography mass spectrometry) at the Institute of Systems Biology (Universiti Kebangsaan Malaysia, Malaysia).

For GLC-MS, chromatographic separation was carried out on a Clarus 600 GC-MS system (PerkinElmer, Shelton, CT, USA) separated with a 30 m × 0.25 mm × 0.25 *μ*m Elite-5MS column (PerkinElmer, USA). The temperature of the oven was set at 40°C and was increased by 5°C/min until it reached 250°C, and the carrier gas was helium at a constant flow of 1 mL/min. The MS parameters used were electron impact mode (EI) following an ionization voltage of 70 eV, an ion source temperature of 200°C, and a scan range of 40–600 Da. The National Institute of Standards and Technology (NIST) (version 2.0, Gaithersburg, MD, USA) database was used for the identification of compounds by exceeding the signal-to-noise ratio (S/N) of 100 and comparing the volatile information based on the compound name. Match and reverse match values below 800 were filtered.

For LC-MS, chromatographic separation was carried out on a Dionex™ UltiMate 3000 UHPLC system (Thermo Scientific) equipped with an Acclaim™ Polar Advantage II C18 column (3 × 150 mm, 3 *μ*m particle size) (Thermo Scientific, USA) by using a 1 *μ*L injection volume. The mobile phase comprised 0.1% formic acid in water (solvent A) and 100% acetonitrile (solvent B), which had a flow rate of 400 *μ*L/min for 22 min. At 0-3 min, 3-10 min, 10-15 min, and 15-22 min, 5% B, 80% B, 80% B, and 5% B were used for the gradient elution, respectively. High-resolution MS analysis was carried out in the positive electrospray ionization mode using a MicrOTOF-Q III (Bruker Daltonik GmbH, Bremen, Germany). A capillary voltage of 4500 V, drying gas flow of 8 L/min, an ion source temperature of 200°C, a nebulizer pressure of 1.2 bar, an end plate offset of −500 V, and a scan range from 50 to 1000 *m*/*z* were used as parameters for the instrument. The METLIN and KNApSAcK databases were used for the identification of compounds by comparing the observed *m*/*z* values with the calculated mass values from previously published data. The abundance of individual compounds was calculated from the percentage of the peak area relative to the total area of all peaks in the chromatograms.

### 2.4. *In Vitro* Evaluation of Antioxidant Properties

#### 2.4.1. Radical Scavenging Activity

The stable radical DPPH (DPPH•) and the stable cation radical ABTS (ABTS•+) were used for measuring free radical scavenging activity. Briefly, the reaction consisted of DPPH• or ABTS•+ solution and the extract (1 mg/mL) at a 1 : 1 ratio. After incubation in the dark for 30 min, the absorbance was read at 517 nm or 734 nm using an EnSpire® Multimode Plate Reader (PerkinElmer, USA) and a UV-VIS spectrophotometer with ascorbic acid (vitamin C) at various concentrations used as a standard. Vitamin C and EGCG were used as positive controls. Radical scavenging activity was expressed as the percent inhibition of the radical calculated by the following equation: % radical scavenging activity = [(Abs of control − Abs of sample) × 100/Abs of control]. The antioxidant capacity was expressed as an EC_50_ value.

#### 2.4.2. Total Phenolic Content

The Folin-Ciocalteu method was used to determine the total phenolic content. In brief, 50 *μ*L of the extract (1 mg/mL) was mixed with 50 *μ*L of a fold-diluted Folin-Ciocalteu phenol reagent. After 20 min, the mixture was neutralized by the addition of 50 *μ*L of a 7.5% (*w*/*v*) Na_2_CO_3_ solution and incubated in the dark for 20 min. Then, the absorbance was measured at 760 nm. Gallic acid was used as a standard for the calibration curve. The total phenolic content was expressed as gallic acid equivalents (GAE/mg of plant extracts).

#### 2.4.3. Total Flavonoid Content

An aluminum chloride colorimetric method was used to measure the total flavonoid content. In brief, 50 *μ*L of the extract (1 mg/mL) was mixed with 150 *μ*L of 95% ethanol, 10 *μ*L of 10% (*v*/*v*) AlCl_3_ solution, and 10 *μ*L of 1 M NaOAc solution. Then, the mixture was incubated in the dark for 40 min and the absorbance was measured at 415 nm. The total flavonoid content was calculated from a calibration curve using quercetin as a standard, and the results are expressed as quercetin equivalents (QE/mg of plant extracts).

### 2.5. *C. elegans* Strains and Culture Condition

The strains N2 (wild type), TK22 (Mev-1[kn1]III), TJ375 (gpIs1[hsp-16-2::GFP]), CF1553 (muls84[pAD76(sod-3::GFP)]), TJ356 (zIs356 [daf-16p::daf-16a/b::GFP+rol-6]), CF1038 (daf-16[mu86]I), BA17 (fem-1[hc17]IV), EU1 (skn-1[zu67]), CL2166 ([pAF15]GST-4p::GFP::NLS), LD1(ldls7), and *Escherichia coli* OP50 were obtained from the Caenorhabditis Genetics Center at the University of Minnesota, USA. All strains were maintained in nematode growth medium (NGM) agar plates at 20°C except for the BA17 strain, which was maintained at 25°C to prevent egg laying. The worms were seeded with living *E. coli* OP50 as a food source. A liquid medium was used for some experiments using S-medium, which was prepared by mixing with *E. coli* OP50 (DO_600_ = 1.0).

Age-synchronized populations of *C. elegans* were obtained by hypochlorite treatment. Worms grown on NGM agar were washed with sterile water and treated with 5 M NaOH and 5% NaOCl at a ratio of 1 : 2 for 8-10 min for lysis and decontamination. The lysate was pelleted by centrifugation (1200 rpm, 2 min) to obtain eggs. The eggs were separated in the pellet layer, and the pellets were washed with sterile water at a ratio of 1 : 2, followed by centrifugation (1200 rpm, 2 min). The upper layer of water was removed, the egg pellet was collected, and eggs were allowed to hatch in M9 buffer [19].

For the treatment group, the toxicity tests were conducted to determine the nontoxic concentration of AO extracts with *E. coli* OP50 (data not shown). Worms were treated with appropriate concentrations of AO extracts: 25, 50, and 100 *μ*g/mL AO hexane extract; 25, 50, and 100 *μ*g/mL AO dichloromethane extract; and 1, 2.5, and 5 *μ*g/mL AO methanol extract. For the control group, worms were treated with 1% (*v*/*v*) DMSO. For the positive control group, worms were treated with 25 *μ*g/mL EGCG in all experiments except the lifespan assay, in which worms were treated with 100 *μ*g/mL EGCG.

### 2.6. Survival Assay under Juglone-Induced Oxidative Stress

To analyze the survival rate under oxidative stress conditions, L1 larvae of wild-type (N2) and transgenic (CF1038, EU1) worms were treated with plant extracts of different concentrations in S-medium for 48 h; each group contained 80 individuals. Each group was treated with 80 *μ*M prooxidant juglone for 24 h. Then, dead and live worms were counted. The worms were considered dead when they failed to respond to a gentle touch with a platinum wire on their bodies [19].

### 2.7. Measurement of Intracellular ROS

To measure the intracellular ROS accumulation in *C. elegans*, L1 larvae of wild-type (N2) and transgenic (CF1038, EU1) worms were treated with plant extracts of different concentrations in S-medium for 48 h; each group comprised of 50-100 individuals. After treatment, the worms were pelleted by centrifugation, added to 50 *μ*M 2,7-dichlorodihydrofluorescein-diacetate (H_2_DCF-DA) solution, and incubated in the dark at 20°C for 1 h. After incubation, worms were paralyzed using 10 mM sodium azide and mounted on a microscopic glass slide. Worms were randomly photographed (30 worms/group) using a BIOREVO BZ-9000 fluorescence microscope (Keyence Deutschland GmbH, Neu-Isenburg, Germany). The relative fluorescence of the whole body was measured and evaluated as the mean fluorescence intensity using ImageJ software (National Institutes of Health, Bethesda, MD).

### 2.8. Quantification of HSP-16.2::GFP, GST-4::GFP, and SOD-3::GFP Expression

The TJ375, CL2166, and CF1553 transgenic worms, which have a gene promoter fused with a GFP reporter, were used to measure the expression of HSP-16.2, GST-4, and SOD-3, respectively. L1 larvae were treated with plant extracts of different concentrations in S-medium at 20°C; each group contained 50-100 worms. The TJ375 and CL2166 transgenic worms were incubated for 72 h and 48 h, respectively. Then, the worms were exposed to a nonlethal dose of 20 *μ*M juglone for 24 h. The CF1553 worms were incubated for 72 h. After that, all worms were submitted to fluorescence microscopy as described before.

### 2.9. Determination of Subcellular Localization of DAF-16 and SKN-1

To determine the localization of the transcription factors DAF-16 and SKN-1, TJ356 and LD1 transgenic strains were used, respectively. L1 larvae were treated with plant extracts of different concentrations in S-medium at 20°C for 72 h; each group contained 50-100 larvae. After incubation, worms were submitted to fluorescence microscopy as described before.

### 2.10. Brood Size Assay

To analyze the potential toxic effect of AO on the reproductive system, brood size was measured. Synchronized N2 worms at the L4 larval stage were sorted and placed one by one on each NGM agar plate. The plates were supplemented with different concentrations of plant extracts in *E. coli* OP50 and incubated at 20°C for 24 h. The adult worms were transferred to fresh medium every day during the reproductive phase to separate them from their progeny. The eggs were counted using a dissecting microscope every day for 4 days to obtain a mean brood size [19].

### 2.11. Body Length and Body Surface Area

To detect the putative effect of dietary restriction by AO, the body lengths and body surface area of the worms were measured. For body length measurement, synchronized and treated worms were analyzed in the same way as in the brood size assay. After treatment, day 1 adult worms were paralyzed by using 10 mM sodium azide and mounted on a microscopic glass slide. Thirty worms were randomly photographed using a 10x objective lens of a brightfield microscope. The body length of worms was analyzed by using the software BZ-II Analyzer (Keyence Corp.) and reported in micrometers.

For body surface area measurement, BA17 transgenic worms in the L1 larval stage were treated with plant extracts of different concentrations in S-medium at 25°C; each group contained 50-100 individuals. The media were changed every second day. On day 8, worms were photographed and the body surface area was measured by ImageJ software (National Institutes of Health, Bethesda, MD).

### 2.12. Quantification of Lipofuscin

To measure the accumulation of the autofluorescent pigment lipofuscin, BA17 transgenic worms were used. Synchronized and treated worms were analyzed in the same way as in the body surface area assay. After treatment until day 16, the worms were paralyzed with 10 mM sodium azide and mounted on a glass slide. Thirty worms were randomly photographed by using a BIOREVO BZ-9000 fluorescence microscope (*λ*ex 360/20 nm, *λ*em 460/38 nm).

### 2.13. Pharyngeal Pumping Rate

To determine a potential age-related decline in muscle function, pharyngeal pumping rates were measured. Synchronization and treatment were carried out as in the brood size assay. L4 larva stage worms were treated with AO extracts for 24 h, except for the control group. Each group contained 30-50 individuals. The adult worms were transferred to fresh medium with treatment every day during the reproductive phase prior to separation from their progeny. After that, the adult worms were transferred to fresh medium with treatment every second day. Pharyngeal pumping was analyzed on days 6, 8, 10, and 12 by counting the pumping frequency of the terminal pharyngeal bulb of each single worm for 60 s. The dissection microscope was used to measure the pumping rate of at least 20 worms from each group. When the worms were crawling on the *E. coli* OP50 lawn, the pumping frequency was recorded and represented as pumps·min^−1^ [19].

### 2.14. Lifespan Assay

To determine the lifespan, wild-type (N2) and transgenic (TK22) worms were used. Synchronization and treatment were carried out as in the brood size assay. Each group contained 30-50 individuals. The worms were counted every day and were presented as a percentage of surviving worms. Worms that failed to respond to a gentle touch with a platinum wire were scored as dead and excluded from the plates. The worms with internally hatched progeny or extruded gonads were scored as censors and discarded from the assay.

### 2.15. Statistical Analysis

Measurements are presented as the mean of three independent runs (mean ± SEM) and performed with GraphPad Prism 6. Statistical comparison between control and treatments was performed by one-way ANOVA following Bonferroni's method (post hoc). Lifespan data were determined by log-rank (Mantel-Cox) tests followed by the Gehan-Breslow-Wilcoxon test. All the experiments were performed at least three times. Differences between the data were considered significant at *p* < 0.05.

## 3. Results

### 3.1. Phytochemical Constituents of AO Hexane and Methanol Extracts

**Figure 1:**
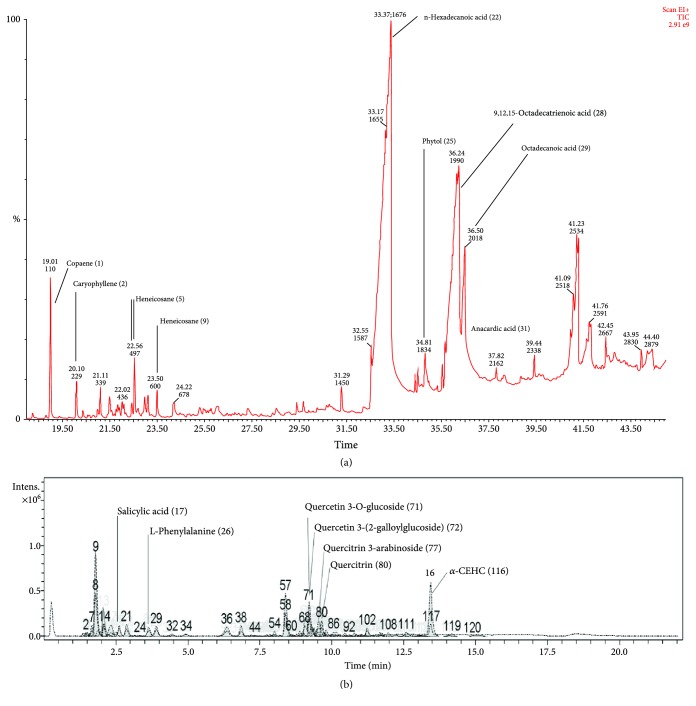
(a) GLC-MS profile of the AO hexane extract. (b) LC-MS run of the AO methanol extract.

The extraction of AO leaves was carried out by using a Soxhlet with different solvents including hexane, dichloromethane, and methanol. The extraction yields were 1.21%, 0.46%, and 15.97%, respectively. GLC-MS and LC-MS were used for the phytochemical analysis of the extracts. Chromatographic peaks were identified by comparing *m*/*z* values of molecular ion peaks in the positive mode [M + H]+ with those in databases. GLC-MS of the hexane extract resulted in more than 50 peaks (Figure 1(a)). Moreover, LC-MS of the methanol extract produced more than 120 peaks (Figure 1(b)) (details in Table S1-2 (supporting information)). We tentatively identified 9 compounds (peak nos. 22, 28, 29, 1, 25, 2, 9, 31, and 5) from the hexane extract as well as 7 compounds (peak nos. 116, 71, 77, 80, 72, 17, and 26) from the methanol extract.

### 3.2. Antioxidant Activities of AO Extracts

The influence of the extraction solvents on the recovery of antioxidant secondary metabolites was studied first (Table 1). The methanol extract exhibited powerful antioxidant activity *in vitro*. When tested in the DPPH and ABTS assays, AO methanol extract effectively scavenged the radical by 90.62% (EC_50_ = 11.32 *μ*g/mL) and 99.36% (EC_50_ = 5.94 *μ*g/mL), respectively (details in Figure S1-2 (supporting information)).

These values are in a similar range as those of known antioxidants, such as EGCG and ascorbic acid. In accordance with the antioxidant activities, high phenolic and flavonoid contents of 160.35 GAE/g dry weight sample and 46.96 QE/g dry weight sample were recorded from the methanol extract.

Moreover, AO hexane extract showed radical scavenging activity by 61.03% (EC_50_ = 11.50 *μ*g/mL), as determined by the ABTS assay. The ABTS assay can measure the scavenging activity of hydrophilic and hydrophobic substances. It is possible that the scavenging activity of the AO hexane extract depends on some hydrophobic bioactive compounds in the AO hexane extract. The combined results agree with a recent study showing that AO extracts contain polyphenols and have antioxidant behavior [14, 20]. However, AO dichloromethane extract did not exhibit antioxidant activity, so this extract was omitted for the subsequent experiments.

**Figure 2:**
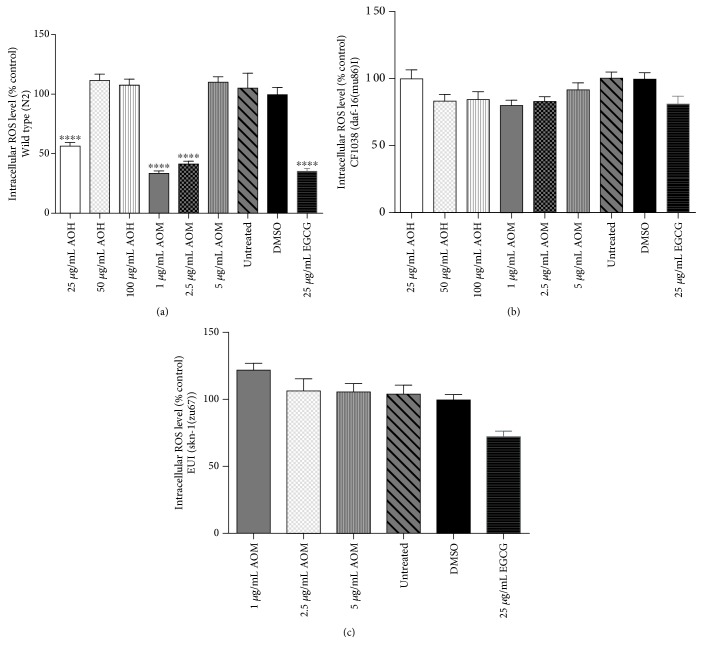
Effect of AO extracts on intracellular ROS in wild-type (a), CF1038 (b), and EU1 (c) worms. AO extract treatment reduced ROS levels in N2 worms when compared to the DMSO control group. Worms were treated with different concentrations of hexane (AOH) and methanol extracts (AOM). DMSO and EGCG were used as the solvent control and positive control groups, respectively. Data are presented as the mean ± SEM (*n* = 80, replicated three times). ^∗^ *p* < 0.05, ^∗∗^ *p* < 0.01, ^∗∗∗^ *p* < 0.001, and ^∗∗∗∗^ *p* < 0.0001, compared to the DMSO control by one-way ANOVA following Bonferroni's method (post hoc).

To analyze the antioxidant activities of AO extracts *in vivo*, the intracellular ROS accumulation levels were measured by using H_2_DCF-DA, which becomes deacetylated by intracellular esterases, emitting fluorescence that correlates with intracellular ROS levels [22].  Figure 2 shows the effect of AO extracts on intracellular ROS, and representative microscopy images from individual worms can be found in the supporting information (Figure S3). The wild-type worms treated with 25 *μ*g/mL hexane extract exhibited a reduced intracellular ROS accumulation of 45.24% (adjusted *p* < 0.0001). Additionally, the wild-type worms treated with 1.0 and 2.5 *μ*g/mL methanol extract had a reduced intracellular ROS accumulation of 7.22% and 58.10%, respectively (adjusted *p* < 0.0001) (Figure 2(a)). The results are in a similar range as the positive control EGCG, which is a well-known antioxidant from green tea [17]. Higher concentrations of both extracts (50 and 100 *μ*g/mL hexane extract, >5 *μ*g/mL methanol extract) were tested; they did not attenuate ROS levels in the worms, possibly due to a prooxidant activity of the plant extracts. However, AO extracts failed to reduce ROS levels in CF1038 (DAF-16 mutant) and EU1 (SKN-1 mutant) worms (Figures 2(b) and 2(c)). These data indicate that the AO extracts at moderate concentrations have *in vitro* and *in vivo* antioxidant activities by attenuating intracellular ROS levels in *C. elegans.* The DAF-16 and SKN-1 pathways appear to be involved.

Moreover, the interfering effect of the extracts on fluorescent measurements was measured by autofluorescent assay. We found that the AO extracts, DMSO, and EGCG had no effect on autofluorescent pigment expression when comparing with untreated control. These data indicate that the fluorescent intensity in the worms was not affected by EGCG, DMSO, and AO extract treatment (Supporting information Figure S6).

### 3.3. AO Extracts Increase the Survival Rate of Nematodes under Oxidative Stress

**Figure 3:**
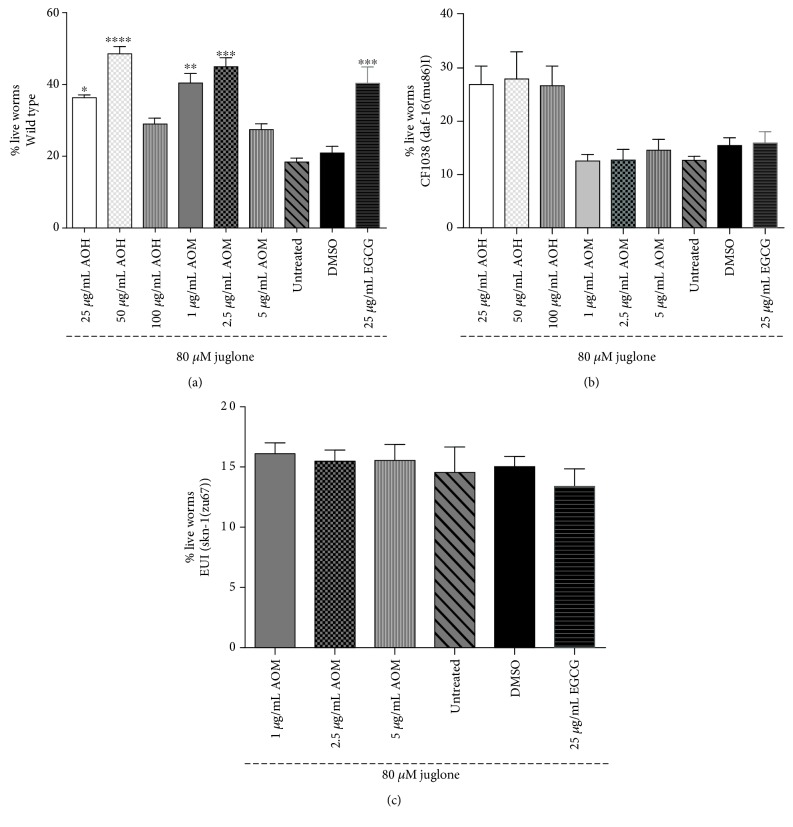
Effect of AO extracts on the survival rate of wild-type (a), CF1038 (b), and EU1 (c) worms under oxidative stress induced by juglone. AO extracts protect against oxidative stress in wild-type *C. elegans* as evidenced by the survival rate of wild-type (N2) worms, which was significantly enhanced after pretreatment with the extracts. However, AO extracts failed to increase the survival rate in CF1038 and EU1 worms. Worms were treated with AO hexane and methanol extracts at different concentrations. DMSO and EGCG were used as the solvent control and positive control groups. Data are presented as the mean ± SEM (*n* = 80, replicated three times). ^∗∗^ *p* < 0.01 and ^∗∗∗^ *p* < 0.001, compared to the DMSO control by one-way ANOVA following Bonferroni's method (post hoc).

To further investigate the antioxidant properties of AO extracts, the survival of nematodes was analyzed under oxidative stress conditions. When treated with 80 *μ*M juglone for 24 h, only 21% of the worms survived. The survival rate for wild-type worms pretreated with 25 and 50 *μ*g/mL hexane extract increased by 36.71% (*p* < 0.05) and 49.15% (*p* < 0.0001), respectively. Moreover, the survival rate for wild-type worms pretreated with 1.0 and 2.5 *μ*g/mL methanol extract increased by 40.89% (*p* < 0.01) and 45.48% (*p* < 0.001), respectively (Figure 3(a)). The survival rate was similar to that of the EGCG-positive control (40.80%). However, higher concentrations of AO extracts (≥100 *μ*g/mL hexane extract and ≥5 *μ*g/mL methanol extract) did not significantly increase the survival rate of the worms under oxidative conditions compared to those of the DMSO reagent control group, possibly due to their prooxidant activity *in vivo* (supporting information Figure S4).

### 3.4. AO Extracts Suppressed the Stress-Induced Expression of HSP-16.2/GFP

**Figure 4:**
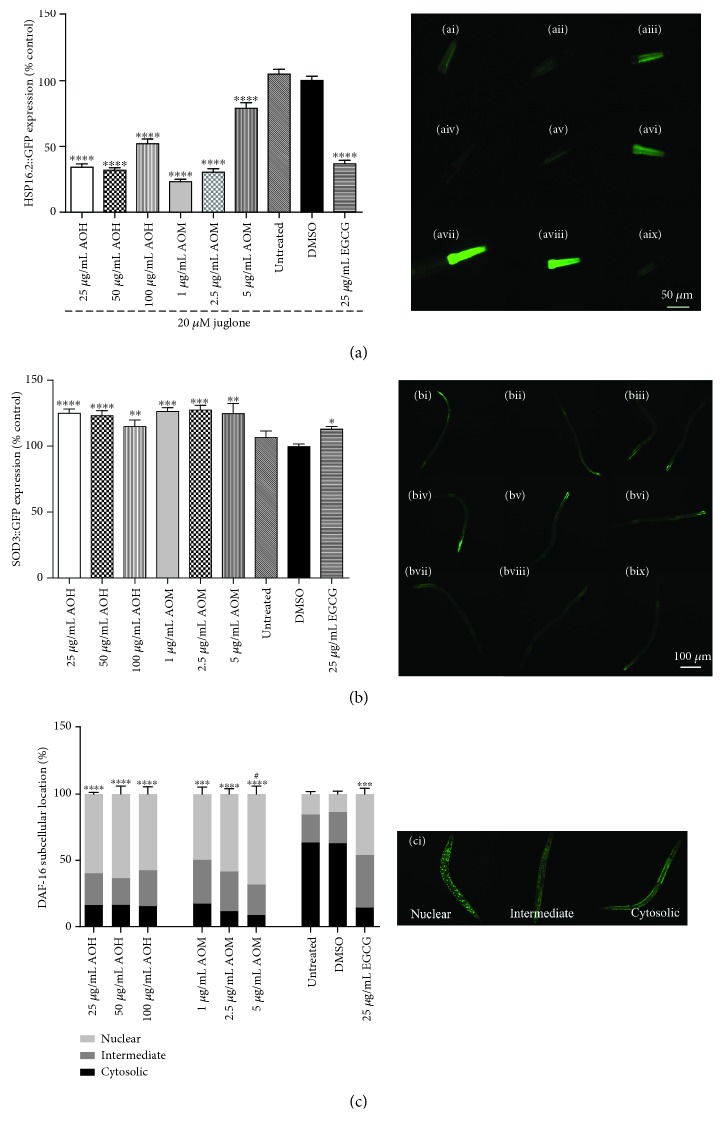
Effect of AO extracts on the expression of stress resistance-related genes and nuclear localization of DAF-16. (a) AO extracts decreased HSP 16.2 expression in mutant TJ375 worms (HSP-16.2::GFP(gplsI)) under oxidative stress induced by juglone. (b) AO extracts increased SOD-3 expression in mutant CF1553 worms ((pAD76) sod-3p::GFP+rol-6), and (c) AO extracts induced a significant translocation of DAF-16::GFP in mutant TJ356 worms (daf-16p::daf-16a/b::GFP+rol-6). (ci) Representative fluorescent images of the subcellular location of DAF-16 in the nucleus, intermediate, and cytosolic regions. (ai–aix) and (bi–bix) Representative pictures of GFP fluorescence in worms treated with 25 *μ*g/mL AOH (ai/bi), 50 *μ*g/mL AOH aii/bii), 100 *μ*g/mL AOH (aiii/biii), 1 *μ*g/mL AOM (aiv/biv), 2.5 *μ*g/mL AOM (av/bv), 5 *μ*g/mL AOM (avi/bvi), untreated control (avii/bvii), DMSO solvent control (aviii/bviii), and 25 *μ*g/mL EGCG (aix/bix). The GFP mean pixel density for each group was calculated from the mean value of the 30 worms that were randomly selected. Data were obtained from three independent experiments and are presented as the mean ± SEM. DMSO and EGCG were used as the solvent control group and positive control group, respectively. ^∗^ *p* < 0.05,^∗∗^ *p* < 0.01, ^∗∗∗^ *p* < 0.001, and ^∗∗∗∗^ *p* < 0.0001, compared to the DMSO control; ^#^ *p* < 0.05 compared to the EGCG-positive control by one-way ANOVA following Bonferroni's method (post hoc).

To determine the mechanism of antioxidant activities, the protective activities of AO extracts against oxidative stress were studied. In nematodes, the promoter for heat shock protein 16.2 (HSP-16.2) was joined with GFP (strain TJ375). Under oxidative stress conditions (20 *μ*M juglone), HSP-16.2 induction was visualized by a high intensity of GFP fluorescence in the head of the transgenic worms. However, when TJ375 worms were pretreated with 25, 50, and 100 *μ*g/mL hexane extract, the fluorescence intensity of GFP was lower than that of the DMSO control group under the same conditions by 64.90%, 67.77%, and 45.86% (*p* < 0.0001), respectively. Moreover, TJ375 worms pretreated with 1.0, 2.5, and 5 *μ*g/mL methanol extract demonstrated fluorescence intensities that were reduced by 78.04%, 68.27%, and 22.32% (*p* < 0.0001), respectively (Figure 4(a)). HSP-16.2 expression under juglone-induced oxidative stress was suppressed in worms by AO extracts similar to 25 *μ*g/mL EGCG (61.91%, *p* < 0.0001). These data indicate that the AO extracts are bioavailable and exhibit *in vivo* antioxidant properties.

### 3.5. AO Extracts Induced the Expression of SOD-3/GFP in the Transgenic *C. elegans*

To further investigate the antioxidant properties of the extracts in the context of antioxidant enzymes, SOD-3 (superoxide dismutase 3) expression was determined in CF1553 worms. Compared to the DMSO control group, the CF1553 worms treated with 25, 50, and 100 *μ*g/mL hexane extract exhibited GFP fluorescence increases of 25.41% (*p* < 0.0001), 23.70% (*p* < 0.0001), and 22.64% (*p* < 0.01), respectively. Moreover, the CF1553 worms treated with 1.0, 2.5, and 5 *μ*g/mL methanol extract also showed GFP fluorescence enhanced by 27.37% (*p* < 0.001), 29.11% (*p* < 0.001), and 24.87% (*p* < 0.01), respectively, similar to the EGCG treatment (Figure 4(b)). These data indicate that the AO extracts can increase the *in vivo* antioxidant effect by inducing antioxidant enzymes.

### 3.6. Influence of AO Extracts on Localization of the DAF-16/FoxO Transcription Factor in *C. elegans*

To investigate whether AO extracts mediate their antioxidant activity through the DAF-16/FoxO pathway, the DAF-16 loss-of-function mutant (CF1038) worms were used in survival assays (Figure 3(b)) and intracellular ROS accumulation (Figure 2(b)) and TJ356 transgenic worms were used in the DAF-16 subcellular localization assay (Figure 4(c)). CF1038 worms pretreated with all concentrations of hexane and methanol extracts did not exhibit an increased survival rate under oxidative stress (Figure 3(b)) nor a decreased intracellular ROS accumulation level when compared to the DMSO control (Figure 2(b)). These data indicate that the AO extracts failed to increase the survival rate under oxidative stress and attenuate intracellular ROS levels in DAF-16 loss-of-function mutants.

When compared to the DMSO control group, the TJ356 worms treated with 25, 50, and 100 *μ*g/mL hexane extract showed an increased level of nuclear location of DAF-16::GFP by 59.73% (*p* < 0.0001), 63.74% (*p* < 0.0001), and 57.45% (*p* < 0.0001), respectively (Figure 4(c)). Moreover, in the TJ356 worms treated with 1.0, 2.5, and 5 *μ*g/mL methanol extract, a similar pattern of nuclear location was observed (nuclear localization 49.68% (*p* < 0.001), 58.74% (*p* < 0.0001), and 68.17% (*p* < 0.0001), respectively). These effects are similar to that of 25 *μ*g/mL EGCG as a positive control (45.95%, *p* < 0.01) (Figure 4(c)). Our data show a strong nuclear localization of DAF-16 compared to the DMSO control group (13.56%). Interestingly, 5 *μ*g/mL methanol extract increased the nuclear location of DAF-16::GFP stronger than EGCG (Figure 4(c)). Taken together, survival assays, intracellular ROS accumulation, and DAF-16 subcellular localization assays strongly suggest that AO extracts mediate antioxidant activity and stress resistance in *C. elegans* via the DAF-16/FoxO pathway.

### 3.7. AO Extracts Increased the Expression of GST-4/GFP in the Transgenic *C. elegans*

**Figure 5:**
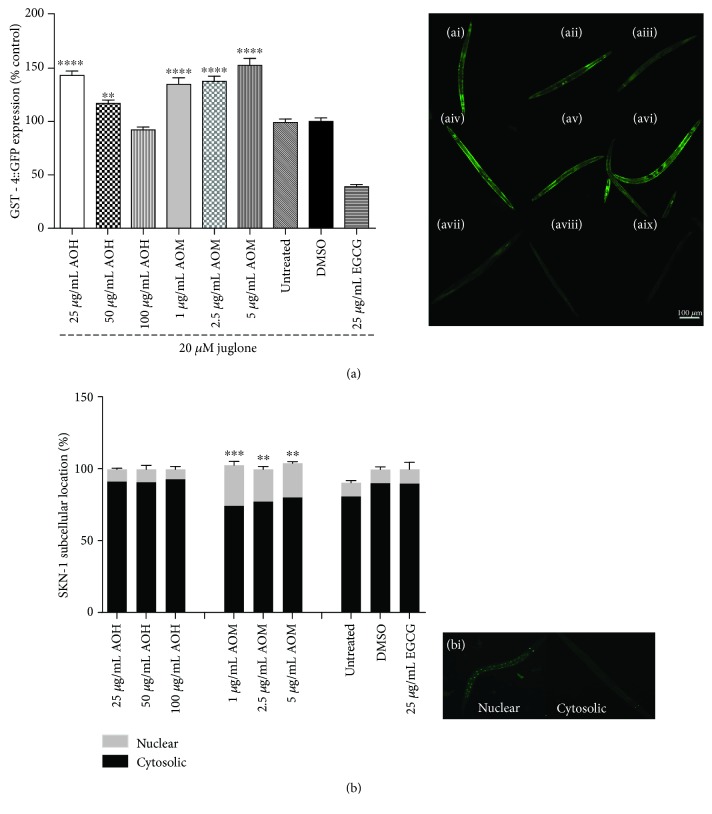
Effect of AO extracts on the expression of stress resistance genes and nuclear localization of SKN-1. (a) AO extract treatment increased GST-4 expression in mutant CL2166 worms ((pAF15)GST-4p::GFP::NLS) under oxidative stress induced by juglone. (b) AO extracts treatment induced a significant translocation of SKN-1::GFP in mutant LD1 worms. (bi) Representative fluorescent images of the subcellular location of SKN-1 in the nucleus and cytosol. (ai–aix) Representative pictures of GFP fluorescence in worms treated with 25 *μ*g/mL AOH (ai), 50 *μ*g/mL AOH (aii), 100 *μ*g/mL AOH (aiii), 1 *μ*g/mL AOM (aiv), 2.5 *μ*g/mL AOM (av), 5 *μ*g/mL AOM (avi), untreated control (avii), DMSO solvent control (aviii), and 25 *μ*g/mL EGCG (aix). The GFP mean pixel density for each group was calculated from the mean value of the 30 worms that were randomly selected. Data were obtained from three independent experiments and presented as the mean ± SEM. DMSO and EGCG were used as the solvent control group and positive control group, respectively. ^∗^ *p* < 0.05, ^∗∗^ *p* < 0.01, ^∗∗∗^ *p* < 0.001, and ^∗∗∗∗^ *p* < 0.0001, compared to the DMSO control by one-way ANOVA following Bonferroni's method (post hoc).

Glutathione is an important antioxidant in cells and is a substrate for glutathione S-transferase. The glutathione S-transferase 4 gene (GST-4) is a target gene in phase II detoxification and is regulated by the SKN-1 signaling pathway [13]. To examine the effect of AO extracts on the SKN-1 pathway, we examined GST-4 expression in CL2166 worms. The CL2166 worms treated with 25 and 50 *μ*g/mL hexane extract exhibited an increased GFP fluorescence intensity (when compared to the DMSO control group) by 41.99% (*p* < 0.0001) and 17.68% (*p* < 0.01), respectively. Moreover, CL2166 worms exposed to 1.0, 2.5, and 5 *μ*g/mL methanol extract showed enhanced GFP fluorescence by 27.38% (*p* < 0.0001), 23.80% (*p* < 0.0001), and 50.19% (*p* < 0.0001), respectively (Figure 5(a)). These data indicate that the antioxidant activity of AO extracts also involves the SKN-1 pathway.

### 3.8. Influence of AO Extracts on Localization of the SKN-1/Nrf-2 Transcription Factor in *C. elegans*

To further investigate whether AO extracts exhibit their antioxidant activity through the SKN-1 signaling pathway, the SKN-1 loss-of-function mutant (EU1) was used in survival assays (Figure 3(c)) and intracellular ROS accumulation (Figure 2(c)).

Pretreatment of these mutants with the methanol extract did not significantly increase the survival rate of the worms under oxidative stress (Figure 3(c)) and nor decrease the intracellular ROS accumulation level (Figure 2(c)) in EU1 worms. These data indicate that the SKN-1 pathway is apparently involved in the oxidative stress response, similar to the DAF-16 pathway.

Moreover, LD1 transgenic worms were subjected to a subcellular localization assay for the transcription factor in SKN-1. The LD-1 worms treated with 1.0, 2.5, and 5 *μ*g/mL methanol extract exhibited an increased nuclear location of SKN-1 :: GFP by 28.49% (*p* < 0.001), 22.55% (*p* < 0.01), and 24.10% (*p* < 0.01), respectively (Figure 5(b)). Whereas the methanol extract induced the nuclear translocation of SKN-1, the hexane extract failed to do so. These findings strongly suggest that AO extracts exert their antioxidant activity and stress resistance effects in *C. elegans* also via the SKN-1 signaling pathway.

### 3.9. AO Extracts Attenuated the Aging Markers

**Figure 6:**
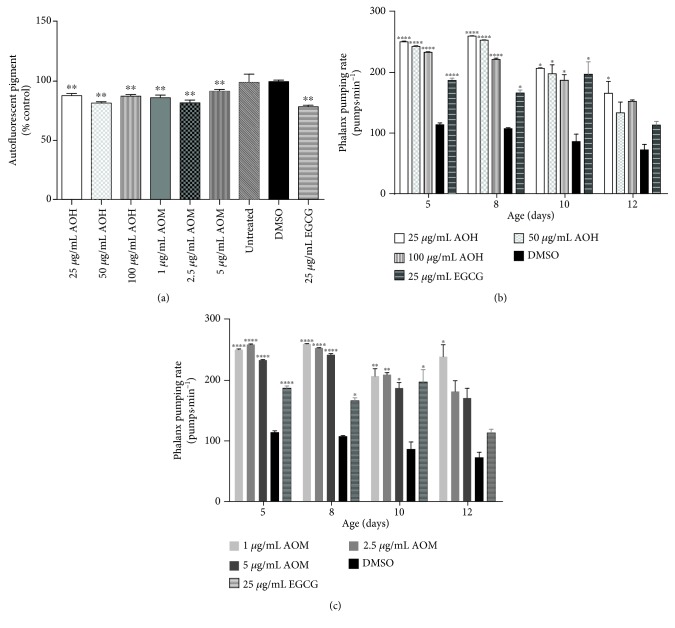
Effect of AO extracts on age-related markers. (a) AO attenuated the autofluorescent pigment lipofuscin in BA17 worms. Autofluorescent granules were measured under the blue wavelength band. Data are presented as the mean ± SEM (*n* = 100). AO extracts improve the pharyngeal pumping rate throughout the *C. elegans* aging process. (b) AO hexane extract. (c) AO methanol extract. DMSO and EGCG were used as the solvent control group and positive control group, respectively. Data are presented as the mean ± SEM (*n* = 30, replicated three times). ^∗^ *p* < 0.05, ^∗∗^ *p* < 0.01, ^∗∗∗^ *p* < 0.001, and ^∗∗∗∗^ *p* < 0.0001, compared to the DMSO control by one-way ANOVA following Bonferroni's method (post hoc).

To investigate the possible influence of AO extracts on aging, the accumulation of the autofluorescent pigment lipofuscin and the pharyngeal pumping rate were measured. The wild-type worms treated with 25, 50, and 100 *μ*g/mL hexane extract had a significantly lower level of lipofuscin (11.96%, *p* < 0.0001; 17.55%, *p* < 0.0001; and 12.62%; *p* < 0.0001, respectively). A similar result was obtained for wild-type worms treated with 1.0, 2.5, and 5 *μ*g/mL methanol extract (Figure 6(a)) (reduction of lipofuscin by 12.25%, *p* < 0.0001; 18.48%, *p* < 0.0001; and 8.39% *p* < 0.01, respectively). These effects are in a similar range as that of 25 *μ*g/mL EGCG (21.58%, *p* < 0.0001).

In another set of experiments, we investigated the influence of AO extracts on muscle function activity, a marker for aging. We determined the pharyngeal pumping rate. On days 5, 8, 10, and 12 of adulthood, wild-type worms were treated with all concentrations of hexane and methanol extracts. These worms displayed a higher pharyngeal pumping rate when compared to worms in the DMSO solvent control group. Moreover, on day 12, the pharyngeal pumping rates of the worms treated with 25 *μ*g/mL hexane extract and 1.0 *μ*g/mL methanol extract were 166% and 222% higher, respectively, than the rate of the worms in the DMSO control group (72%) (*p* < 0.05) (Figures 6(b) and 6(c)). These data (lipofuscin accumulation and pharyngeal pumping rates) indicate that AO extracts apparently have an antiaging effect.

### 3.10. AO Extracts Did Not Interfere with Brood Size, Body Length, and Body Surface Area of *C. elegans*

Aging can be influenced by dietary restriction (DR). We carried out a number of experiments to determine whether AO extracts could exhibit DR.

To investigate a potential interfering effect of AO extracts on DR, brood size and body lengths of the nematodes were measured. Brood size and body length in wild-type worms were not affected by different concentrations of hexane, dichloromethane, and methanol extracts (supporting information: Table S3 and Figure S5). These data indicated that the effects of AO extracts were not caused by DR.

To investigate an interfering effect of AO extracts on the body size of worms, the body surface area was measured. On day 8, the body surface area of worms did not show to be significantly different when compare to that of the DMSO control group. These data indicated that the improvement of pharyngeal pumping rate affected by AO extracts were not interfered by the body size of the worms. (Supporting information Figure S5c).

### 3.11. AO Extracts Extended the Mean Lifespan of Wild-Type *C. elegans*

**Figure 7:**
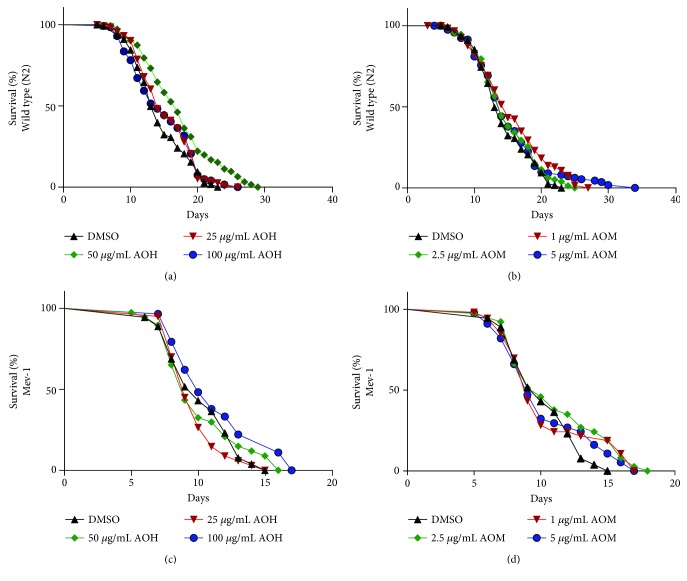
Effect of AO extracts on the lifespan of wild-type (a, b) and Mev-1 mutant (c, d) strain worms. Survival curves of the worms at 20°C on the plate treated with AO hexane extracts (a, c) and methanol extracts (b, d) at different concentrations. Survival plots were drawn by GraphPad Prism 6.0.

To study whether the AO extracts can influence longevity, we analyzed their influence on the lifespan of the wild-type (N2) and Mev-1 mutants (TK22) (a mutation in succinate dehydrogenase cytochrome b causes oxidative stress and short lifespan) worms [23]. The hexane extract (50 *μ*g/mL) increased the mean lifespan of the N2 worms by 20.31% (*p* < 0.001) (Figure 7(a)). Moreover, 1 *μ*g/mL methanol extract induced a mean lifespan increase of the N2 worms by 3.36% (*p* < 0.01) (Figure 7(b)). However, AO hexane and methanol extracts failed to extend the mean lifespan of TK22 worms (Figure 7(c) and 7(d)) (details in Table S4 (supporting information)). These data indicated that AO extracts may influence longevity via the DAF-16 pathway.

## 4. Discussion

Natural compounds can represent novel antiaging agents, and numerous studies have reported correlations between natural antioxidant compounds and antiaging capacities [17, 18]. This is the first report describing the antiaging potential and oxidative stress resistance properties of AO leaf extracts observed in *C. elegans*.

In this study, we observed that AO extracts can effectively protect *C. elegans* against severe oxidative stress and attenuate intracellular ROS levels at moderate concentrations. In contrast, the higher concentrations of AO extracts failed. It is possible that AO contains anacardic acid, which was reported to be toxic to melanoma cells, bacteria, and insects at high doses [21, 24, 25]. We assumed that the plant extract might act as a pro-oxidant and need an optimal concentration to protect and decrease the ROS level in the worms [26].

HSPs represent a family of proteins involved in the sensor of oxidative stress function. In *C. elegans*, HSP-16.2 plays a key role in protecting against oxidative stress and ROS [27]. We observed that AO extracts have a protective effect against oxidative stress because they reduce intracellular ROS accumulation and counteract the activity of juglone (observed via the reduction of HSP-16.2 expression). These abilities were similar to effects of the antioxidant agents such as EGCG [17] and anthocyanin-rich purple wheat extracts [28].

The insulin/IGF-1 signaling (IIS) pathway is a well-known longevity pathway in *C. elegans*, which modulates the expression of stress response, metabolism, and longevity through the DAF-16/FoxO transcription factor and its downstream targets [7, 8]. Under normal conditions, DAF-16/FoxO remains inactive in the cytosol until environmental conditions such as stress or certain ligands stimulate DAF-16/FoxO translocation from the cytoplasm to the nucleus, leading to the expression of various genes that contribute to stress response [9]. SOD-3 is an antioxidant enzyme that is activated by DAF-16; it mediates O_2_•− scavenging and balancing of ROS [19]. We observed that AO hexane and methanol extracts affected SOD-3 expression and stress resistance in *C. elegans* via the DAF-16/FoxO pathway. These effects are similar to those of other polyphenols such as an anthocyanin-rich extract of purple wheat [29], an anthocyanin-rich extract of acai [19], and chlorophyll [30] which have also been shown to activate the DAF-16/FoxO pathway in *C. elegans*.

Another pathway that is involved in lifespan and antioxidant pathways is the SKN-1/Nrf-2 signaling pathway. The SKN-1 gene is a well-known stress response gene and is involved in longevity in *C. elegans* [12, 13]. Glutathione (GSH) protects against acute oxidative stress conditions [12]. We observed that the methanol extract affected GST-4 expression and stress resistance in *C. elegans* via the SKN-1 signaling pathway. These effects are similar to some natural products including peptides from sesame and rose essential oils [31, 32]. Although the AO hexane extract did not affect SKN-1 nuclear localization, it induced GST-4 expression. It is possible that GST-4 is activated by another transcription factor. These data were supported by Detienne et al., showing that not only SKN-1 but also EOR-1, which is a transcription factor mediating the effects of the epidermal growth factor (EGF) pathway, can activate GST-4 [33].

In *C. elegans*, muscle function declines in terms of pharyngeal pumping function; pigment accumulation (lipofuscin) is another well-known biomarker of aging. Importantly, hexane and methanol extracts could reduce the level of lipofuscin and improved the pharyngeal pumping rate in late adult worms, which had already been reported for EGCG [17, 34], anthocyanins [19], chlorophyll [30], caffeic acid, quercetin, and kaempferol [18, 35]. AO extracts showed antiaging properties. We found that the worms that were treated with 50 *μ*g/mL hexane extract and 1 *μ*g/mL methanol extract had a significantly longer mean lifespan than wild-type worms. However, AO extracts failed to extend the mean lifespan of Mev-1 mutant worms (TK22), which showed a shortened lifespan. These effects were similar to those seen in some other natural products such as chlorophyll [30] and natural lignans from *Arctium lappa* [26], indicating that the lifespan extension effect is likely not based on the oxidative stress resistance and antioxidant effect alone. This result strongly suggests that endogenous signaling pathways other than a direct antioxidant mechanism are involved. Stress resistance and lifespan extension are mostly dependent on the DAF-16/FoxO-dependent pathway [8]. AO extracts can increase both DAF-16/FoxO and SKN-1 gene expression, which belong to the insulin/IGF-1 signaling (IIS) longevity pathway in *C. elegans*. Thus, the transcription factors DAF-16 and SKN-1 are part of the modulators for lifespan extension in *C. elegans*.

To our knowledge, this is the first report about the bioactive compounds (flavonoid glycoside) in AO leaf extracts. Flavonoid-rich plant extracts and quercetin can extend the lifespan, reduce lipofuscin accumulation, and protect worms against oxidative stress by reducing internal oxidative stress and intracellular ROS in *C. elegans* [18, 35, 36]. These data support our result that AO extracts modulate oxidative stress resistance and lifespan extension in *C. elegans*.

Moreover, phytochemical analysis shows that AO hexane extract contains palmitic acid, *α*-linolenic acid, and *β*-caryophyllene. Previous works have reported that *α*-linolenic acid can recover pharyngeal pumping and increase lifespan in *C. elegans* via the SKN-1 signaling pathway [37, 38] and *β*-caryophyllene can modulate the stress response by reducing intracellular free radical levels and influencing feeding behavior and the pharyngeal pumping rate, as well as reducing intestinal lipofuscin levels and increasing the lifespan in *C. elegans* via SIR-2.1, SKN-1, and DAF-16 [39]. These findings suggest that AO extracts enhance the oxidative stress resistance through the DAF-16/FoxO and SKN-1/Nrf2 signaling pathways. Since it is known that healthspan and lifespan effects are highly correlated [26], AO extracts may mediate both effects via the DAF-16/FoxO and SKN-1/Nrf2 signaling pathways. However, further studies are needed to elucidate the underlying mechanisms of AO extracts on the lifespan extension of *C. elegans*.

## 5. Conclusions

In summary, the AO leaf extracts demonstrated oxidative stress resistance properties via the DAF-16/FoxO and SKN-1/Nrf-2 signaling pathways. AO leaf extracts also have effect on antiaging and lifespan extension in *C. elegans*. Further studies are needed to clarify the underlying mechanisms of AO extracts on the lifespan extension of *C. elegans* and *in vivo* tests with more complex model organisms.

## Figures and Tables

**Table 1:** Total phenolic content, total flavonoid content, and free radical scavenging capacity of AO extracts.

Extract	Total phenolics (mgGAE/g)^∗∗^	Total flavonoids (mgQE/g)^∗∗^	DPPH scavenging assay		ABTS scavenging assay	
			% radical scavenging activity^∗^	EC_50_ (*μ*g/mL)	% radical scavenging activity^∗^	EC_50_ (*μ*g/mL)
AOH	27.00 ± 2.12	2.26 ± 0.14	0.33 ± 1.31	—	61.03 ± 2.70	11.50 ± 3.85
AOD	12.70 ± 2.13	0.17 ± 0.36	0.05 ± 3.75	—	42.93 ± 8.13	74.04 ± 9.83
AOM	160.35 ± 0.83	46.96 ± 0.09	90.62 ± 0.64	11.32 ± 0.45	99.36 ± 3.29	5.94 ± 1.03
Vitamin C	—	—	—	7.91 ± 1.34	—	4.76 ± 0.71
EGCG	—	—	—	6.89 ± 0.33	—	2.59 ± 0.40

AOH: 1 mg/mL AO hexane extract; AOD: 1 mg/mL AO dichloromethane extract; AOM: 1 mg/mL AO methanol extract; ^∗^of 1 mg/mL extract; ^∗∗^dry weight sample; values are expressed as the mean ± SD (*n* = 3).

## Data Availability

The data used to support the findings of this study are included within the article and supplementary information file.

## Abbreviations

AO: *Anacardium occidentale L.*
AOH: AO hexane extract
AOD: AO dichloromethane extract
AOM: AO methanol extract
DAF-16/FoxO: Forkhead box protein O
SKN-1/Nrf-2: Nuclear factor erythroid 2-related factor 2
SOD-3: Superoxide dismutase-3
GST-4: Glutathione S-transferase 4
HSP-16.2: Heat shock protein-16.2
ROS: Reactive oxygen species
H2DCF-DA: 2,7-Dichlorofluorescein diacetate
Juglone: (5-Hydroxy-1,4-naphthoquinone)
ABTS: 2,2-Azino-bis(3-ethylbenzothiazoline-6-sulfonic acid)
DPPH: Diammonium salt, 2,2-diphenyl-1-picrylhydrazyl
DMSO: Dimethyl sulfoxide
EGCG: Epigallocatechin gallate
GC-MS: Gas chromatography mass spectrometry
LC-MS: Liquid chromatography mass spectrometry
VCEAC: Vitamin C equivalent antioxidant capacity
GAE: Gallic acid equivalents
QE: Quercetin equivalents
NaOH: Sodium hydroxide
NaOCl: Sodium hypochlorite.

## Supplementary Materials

Supplementary Materials Supplementary Figure S1: ABTS radical scavenging activity of AOH (4.69-600 *μ*g/mL) (a), AOD (37.5-600 *μ*g/mL) (b), AOM (2.5-40 *μ*g/mL) (c), EGCG, and vitamin C (0.31-20 *μ*g/mL) (d). Supplementary Figure S2: DPPH radical scavenging activity of AOM (1.25-40 *μ*g/mL) (a) and EGCG and vitamin C (0.31-20 *μ*g/mL) (b). Supplementary Figure S3: representative pictures of DCFDA fluorescence in worms treated with 25 *μ*g/mL AOH (a), 50 *μ*g/mL AOH (b), 100 *μ*g/mL AOH (c), 1 *μ*g/mL AOM (d), 2.5 *μ*g/mL AOM (e), 5 *μ*g/mL AOM (f), untreated control (g), DMSO solvent control (h), and 25 *μ*g/mL EGCG (i). Supplementary Figure S4: prooxidant effect of AO extracts on intracellular ROS in wild-type worms. High concentrations of AO extract (150 and 200 *μ*/mL AOH; 10 and 20 *μ*g/mL AOM) treatment increased ROS levels in N2 worms when compared to the DMSO control group. Data are presented as the mean ± SEM (*n* = 80, replicated three times). ^∗^ *p* < 0.05, ^∗∗^ *p* < 0.01, ^∗∗∗^ *p* < 0.001, and ^∗∗∗∗^ *p* < 0.0001, compared to the DMSO control by one-way ANOVA following Bonferroni's method (post hoc). Supplementary Table 1: proposed phytochemical constituents in the AO hexane extract using GC-MS. Supplementary Table 2: proposed phytochemical constituents in the AO methanol extract using LC-MS. Supplementary Table 3: effect of AO extracts on markers of aging and development in *C. elegans*. Supplementary Figure S5: (a) brood size (wt), (b) body length (wt), and (c) body surface area (BA17) of worms after AO extract treatment. Treatment with AO had no effect on egg laying activity, body length, and body surface area. The results are expressed as the mean ± SEM from three independent experiments (*n* = 30). Treatment groups are compared to the DMSO control by one-way ANOVA following Bonferroni's method (post hoc). Supplementary Figure S6: AO extract treatment had no effect on autofluorescent pigment expression. The fluorescent intensity in the worms was not affected by EGCG, DMSO, and AO extract treatment. Data are presented as the mean ± SEM (*n* = 100), compared to the untreated control by one-way ANOVA following Bonferroni's method (post hoc). Supplementary Table 4: results and statistical analyses of the AO extract-treated *C. elegans* in lifespan assay.
