# Supplementary material for: Lifespan Extending and Oxidative Stress Resistance Properties of a Leaf Extracts from Anacardium occidentale L. in Caenorhabditis elegans
Source: Oxid Med Cell Longev. 2019 Jun 2;2019:9012396. doi: 10.1155/2019/9012396 (PMC6589224; doi:10.1155/2019/9012396)
Supplement: Supplementary Materials — Supplementary Figure S1: ABTS radical scavenging activity of AOH (4.69-600 μg/mL) (a), AOD (37.5-600 μg/mL) (b), AOM (2.5-40 μg/mL) (c), EGCG, and vitamin C (0.31-20 μg/mL) (d). Supplementary Figure S2: DPPH radical scavenging activity of AOM (1.25-40 μg/mL) (a) and EGCG and vitamin C (0.31-20 μg/mL) (b). Supplementary Figure S3: representative pictures of DCFDA fluorescence in worms treated with 25 μg/mL AOH (a), 50 μg/mL AOH (b), 100 μg/mL AOH (c), 1 μg/mL AOM (d), 2.5 μg/mL AOM (e), 5 μg/mL AOM (f), untreated control (g), DMSO solvent control (h), and 25 μg/mL EGCG (i). Supplementary Figure S4: prooxidant effect of AO extracts on intracellular ROS in wild-type worms. High concentrations of AO extract (150 and 200 μ/mL AOH; 10 and 20 μg/mL AOM) treatment increased ROS levels in N2 worms when compared to the DMSO control group. Data are presented as the mean ± SEM (n = 80, replicated three times). ∗ p < 0.05, ∗∗ p < 0.01, ∗∗∗ p < 0.001, and ∗∗∗∗ p < 0.0001, compared to the DMSO control by one-way ANOVA following Bonferroni's method (post hoc). Supplementary Table 1: proposed phytochemical constituents in the AO hexane extract using GC-MS. Supplementary Table 2: proposed phytochemical constituents in the AO methanol extract using LC-MS. Supplementary Table 3: effect of AO extracts on markers of aging and development in C. elegans. Supplementary Figure S5: (a) brood size (wt), (b) body length (wt), and (c) body surface area (BA17) of worms after AO extract treatment. Treatment with AO had no effect on egg laying activity, body length, and body surface area. The results are expressed as the mean ± SEM from three independent experiments (n = 30). Treatment groups are compared to the DMSO control by one-way ANOVA following Bonferroni's method (post hoc). Supplementary Figure S6: AO extract treatment had no effect on autofluorescent pigment expression. The fluorescent intensity in the worms was not affected by EGCG, DMSO, and AO extract treatment. Data are presented as t [file 9012396.f1.pdf]

Supplementary Materials

Lifespan extending and oxidative stress resistance properties of a leaf extracts from *Anacardium occidentale* L. in *Caenorhabditis elegans*

Chatrawee Duangjan,[1,2,3] Panthakarn Rangsinth,[1,2,3] Xiaojie Gu,[2,4] Michael Wink,[2]* and Tewin Tencomnao,[1,3]*

*** Corresponding authors**

[1]Graduate Program in Clinical Biochemistry and Molecular Medicine, Department of Clinical Chemistry, Faculty of Allied Health Sciences, Chulalongkorn University, Bangkok 10330, Thailand

[2]Institute of Pharmacy and Molecular Biotechnology, Im Neuenheimer Feld 364, Heidelberg University, 69120 Heidelberg, Germany. Wink@uni-heidelberg.de

[3]Age-Related Inflammation and Degeneration Research Unit, Department of Clinical Chemistry, Faculty of Allied Health Sciences, Chulalongkorn University, Bangkok, 10330, Thailand. tewin.t@chula.ac.th

[4]Department of Biotechnology, School of Environmental and Chemical Engineering, Dalian Jiaotong University, Dalian 116028, China

Supplementary Table and Figure

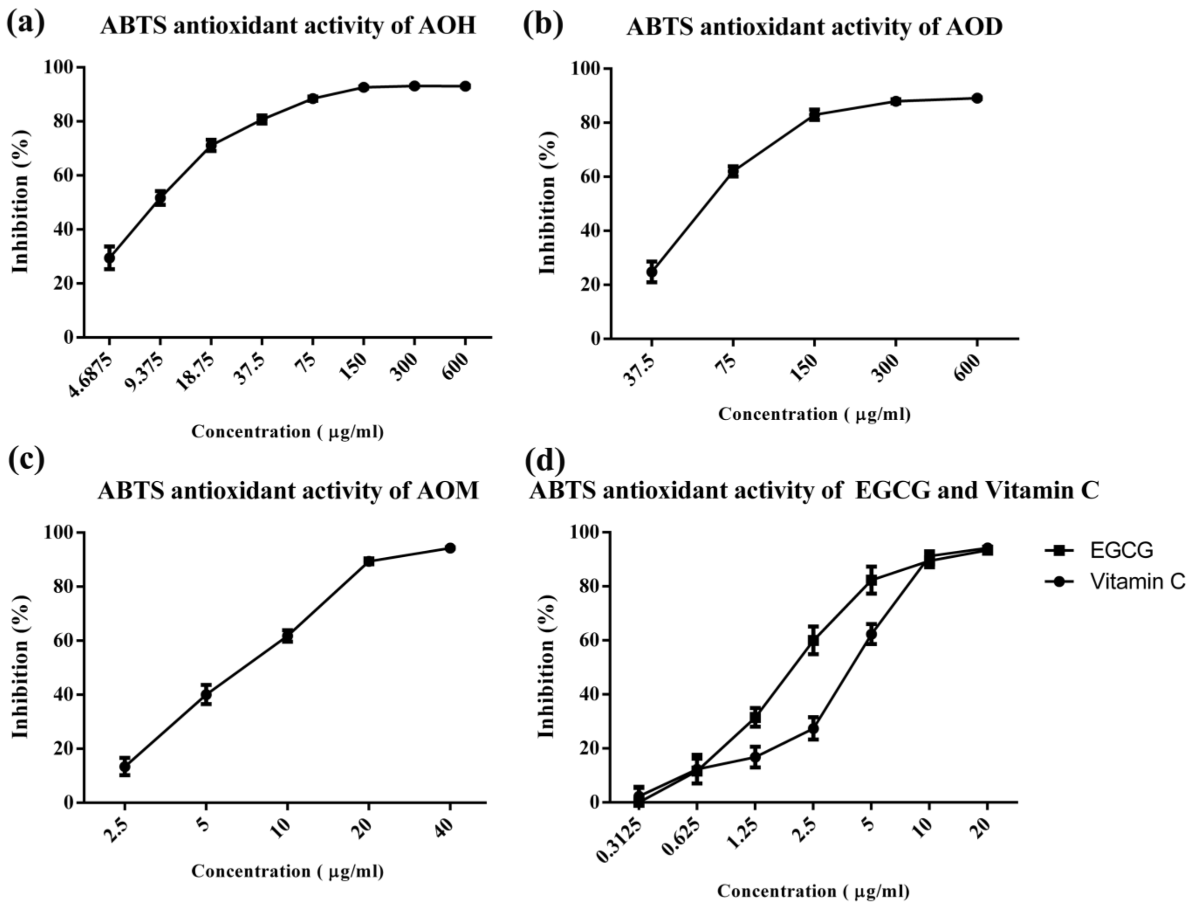

Supplementary Figure S1. ABTS radical scavenging activity of AOH (4.69-600 μg/mL)**(a),** AOD (37.5-600 μg/mL)**(b)**, AOM (2.5-40 μg/mL)**(c)**, EGCG and vitamin C (0.31-20 μg/mL)**(d)**.

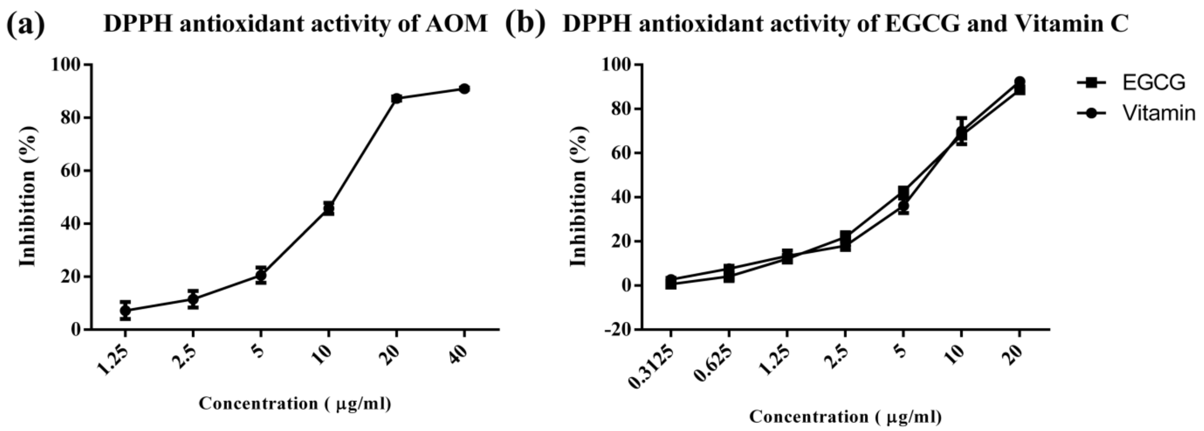

Supplementary Figure S2. DPPH radical scavenging activity of AOM (1.25-40 µg/mL)**(a)**, EGCG and vitamin C (0.31-20 µg/mL)**(b)**.

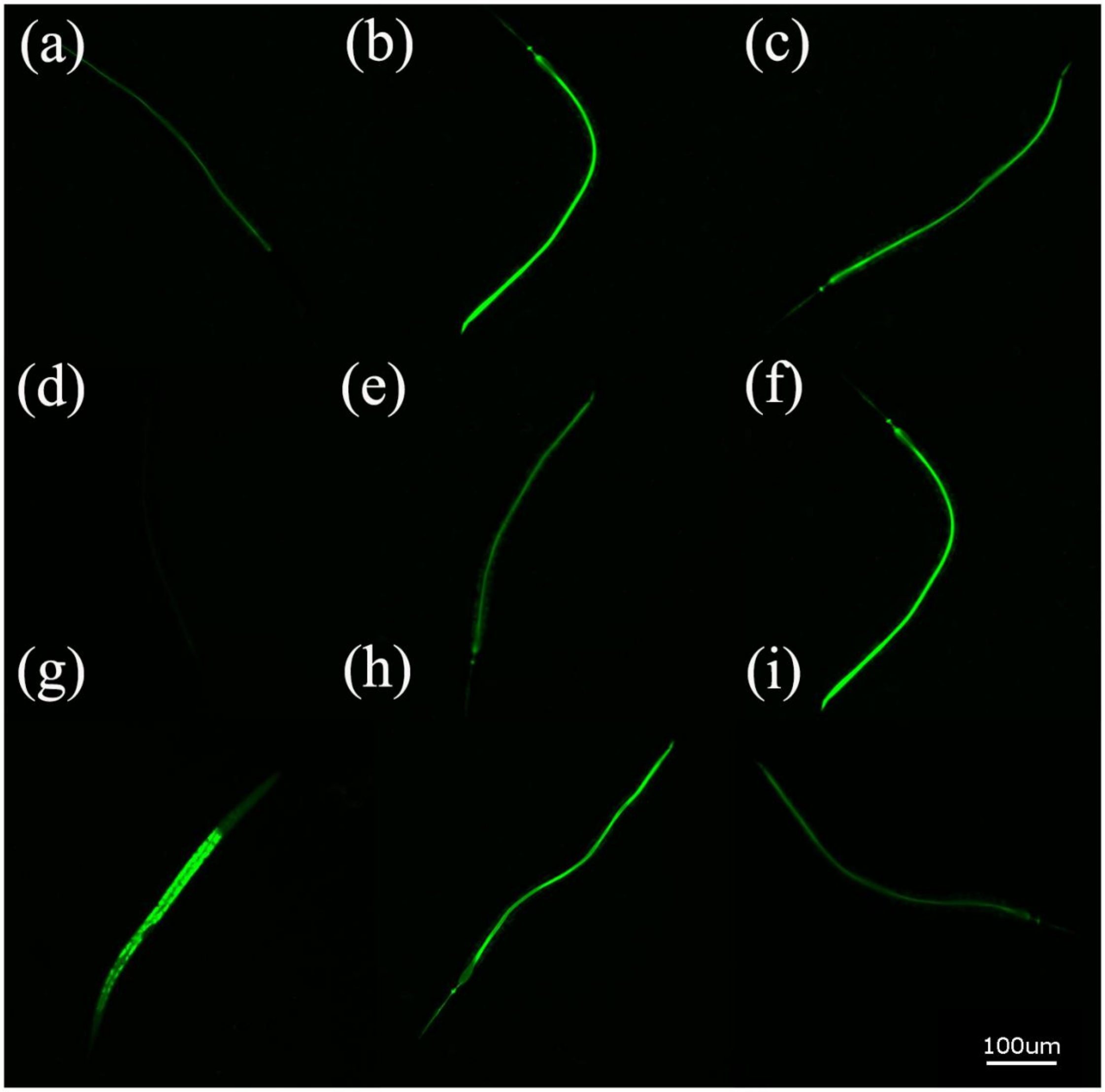

Supplementary Figure S3. Representative pictures of DCFDA fluorescence in worms treated with 25 µg/mL AOH **(a)**; 50 µg/mL AOH **(b)**; 100 µg/mL AOH **(c)**; 1 µg/mL AOM **(d)**; 2.5 µg/mL AOM **(e)**; 5 µg/mL AOM **(f)**; Untreated control **(g)**; DMSO solvent control **(h)**; and 25 µg/mL EGCG **(i)**.

AO extracts showed lower levels of ROS in N2 worms when compared to the DMSO control group. DMSO and EGCG were used as a solvent control and positive control group, respectively.

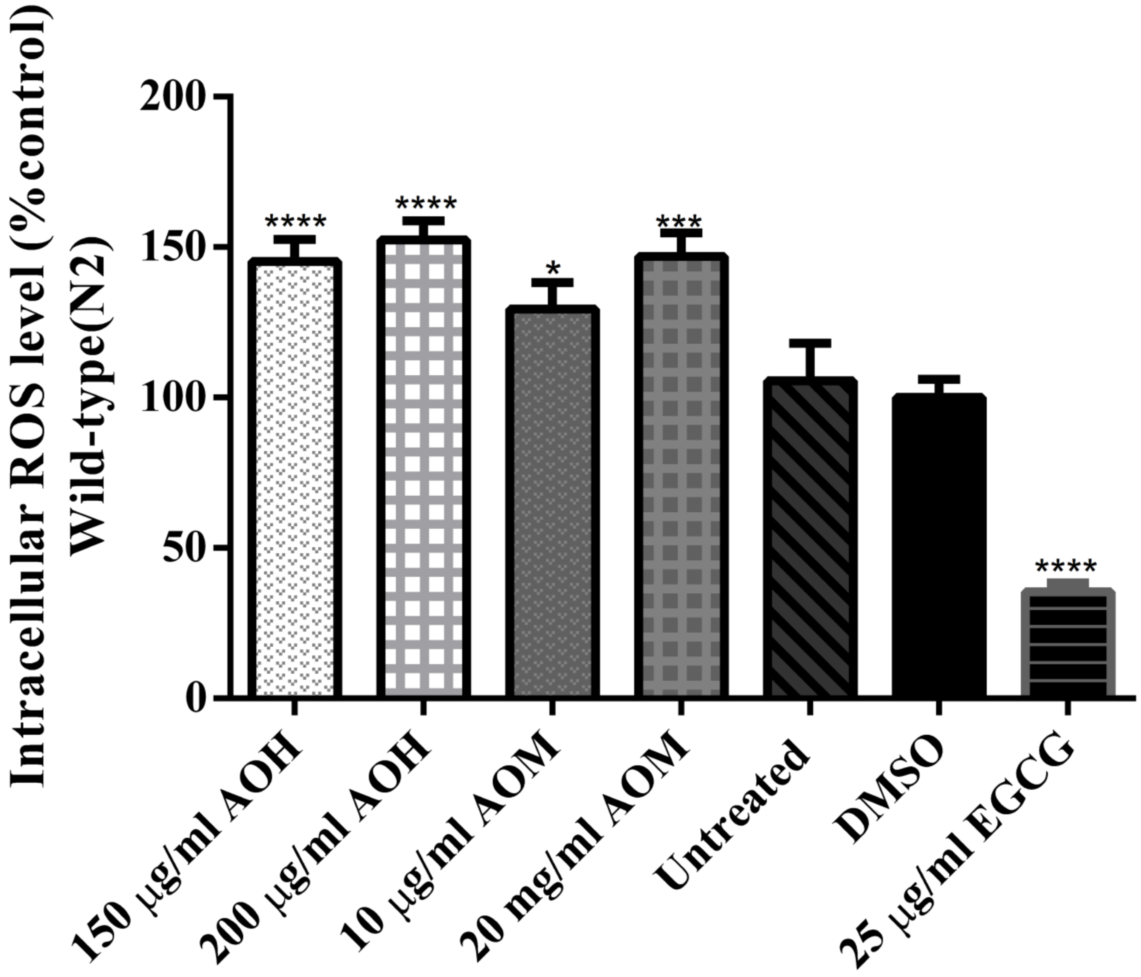

Supplementary Figure S4. Pro-oxidant effect of AO extracts on intracellular ROS in wild-type worms. High concentrations of AO extracts (150 and 200 μ/mL AOH; 10 and 20 μg/mL AOM) treatment increased ROS levels in N2 worms when compared to the DMSO control group. Data are presented as the mean ± SEM (n = 80, replicated three times). *$p < 0.05$, **$p < 0.01$, ** *$p < 0.001$ and ****$p < 0.0001$, compared to the DMSO control by one-way ANOVA following Bonferroni's method (post hoc).

Supplementary Table S1. Proposed phytochemical constituents in the AO hexane extract using GC-MS

Peak No.	Rt(min)	Area (%)	Proposed compound	Match	Prob.
1	19.0080	1.565	Copaene	926	33.1000
2	20.0990	0.457	Caryophyllene	942	32.7000
5	22.0240	0.175	Heneicosane	824	11.4000
9	23.5000	0.389	Heneicosane	901	15.9000
22	33.3660	41.807	n-Hexadecanoic acid or Palmitic acid	893	68.8000
25	34.8150	0.801	Phytol	824	23.1000
28	36.2450	19.606	9,12,15-Octadecatrienoic acid, (Z,Z,Z)- or α-Linolenic acid	833	40.6000
29	36.5020	6.914	Octadecanoic acid or Stearic acid	875	87.5000
31	37.8220	0.345	Monounsaturated anacardic acid	788	9.2000

Library: MAINLIB

Supplementary Table S2. Proposed phytochemical constituents in the AO methanol extract using LC-MS

Peak No.	Rt (min)	[M + H]+ (m/z)	Area (%)	Proposed compound	Theoretical mass	Mass error (ppm)
17	2.3	139.0395	2.0142	Salicylic acid	138.0320	3
26	3.7	166.0856	1.4076	L-Phenylalanine	165.0790	3
71	9.2	465.1032	4.0410	Quercetin 3-O-glucoside	464.0960	0
72	9.3	617.1136	1.3883	Quercetin 3-(2-galloylglucoside)	616.1060	0
77	9.6	435.0922	2.0603	Quercetin 3-arabinoside	434.0850	0
80	9.7	449.1078	1.6803	Quercitrin / Kaempferol-7-O-glucoside	448.1010	0
116	13.4	279.1587	7.4592	α-CEHC,tocopherols	278.1528	1

Database: METLIN (CA, USA) and KNApSAcK Keyword Search Web Version 1.000.01

Supplementary Table 3. Effect of AO extracts on markers of aging and development in *C. elegans*

Extract	Brood size(mean egg lay)	Body length (mean length (μm))
DMSO reagent control	245.5 ± 33.50	1517 ± 18.97
25 μg/ml AOH	245.3 ± 22.34	1422 ± 27.94
50 μg/ml AOH	235.0 ± 38.23	1423 ± 34.87
100 μg/ml AOH	238.3 ± 14.68	1506 ± 15.70
25 μg/ml AOD	270.3 ± 10.22	1502 ± 13.74
50 μg/ml AOD	199.3 ± 8.179	1469 ± 25.86
100 μg/ml AOD	270.8 ± 3.198	1468 ± 15.16
1 μg/ml AOM	247.8 ± 12.24	1591 ± 19.79
2.5 μg/ml AOM	275.5 ± 5.50	1444 ± 18.23
5 μg/ml AOM	220.0 ± 11.27	1494 ± 17.70

AOH; AO hexane extract, AOD; AO dichloromethane extract, AOM; AO methanol extract
(Results are means ± SEM)

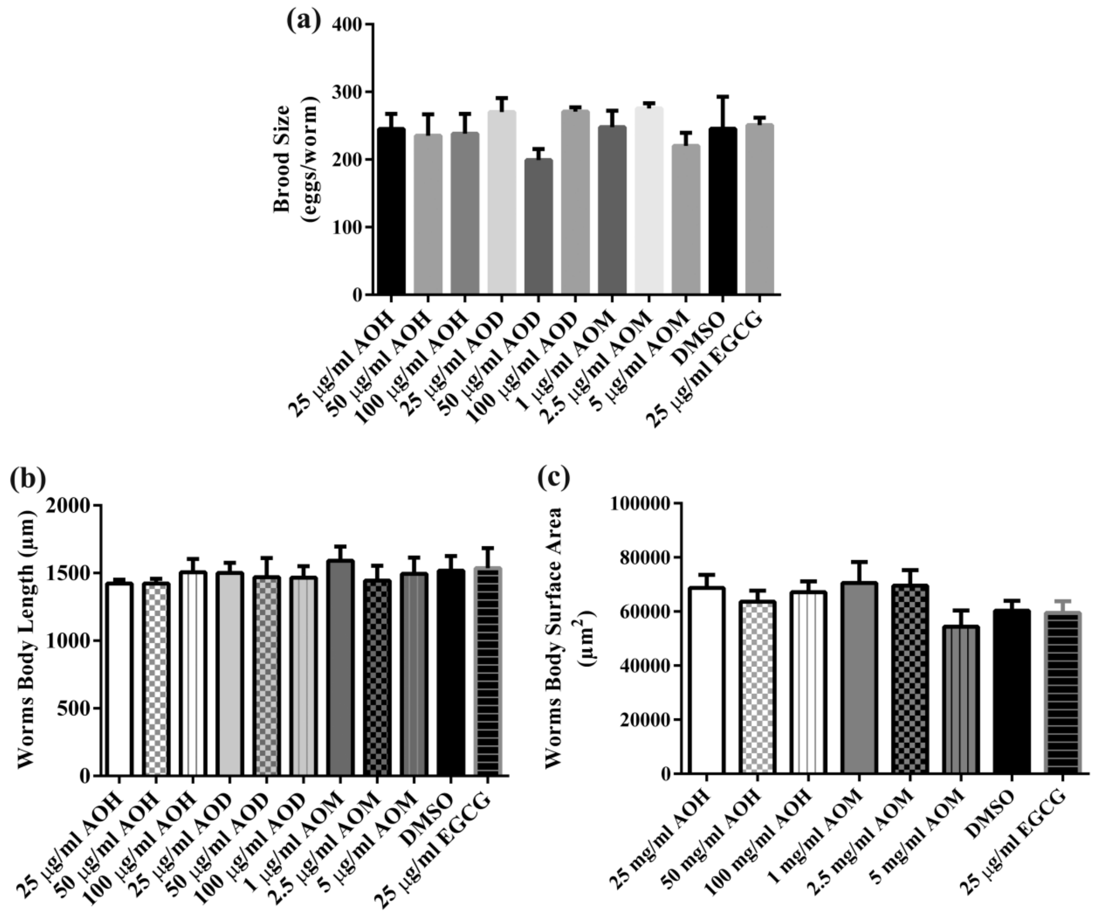

Supplementary Figure S5. (a) Brood size (wt), **(b)** body length (wt) and **(c)** body surface area (BA17) of worms after AO extracts treatment. Treatment with AO had no effect on egg laying activity, body length and body surface area. The results are expressed as the mean ± SEM from three independent experiments (n = 30). Treatment groups are compared to the DMSO control by one-way ANOVA following Bonferroni's method (post hoc).

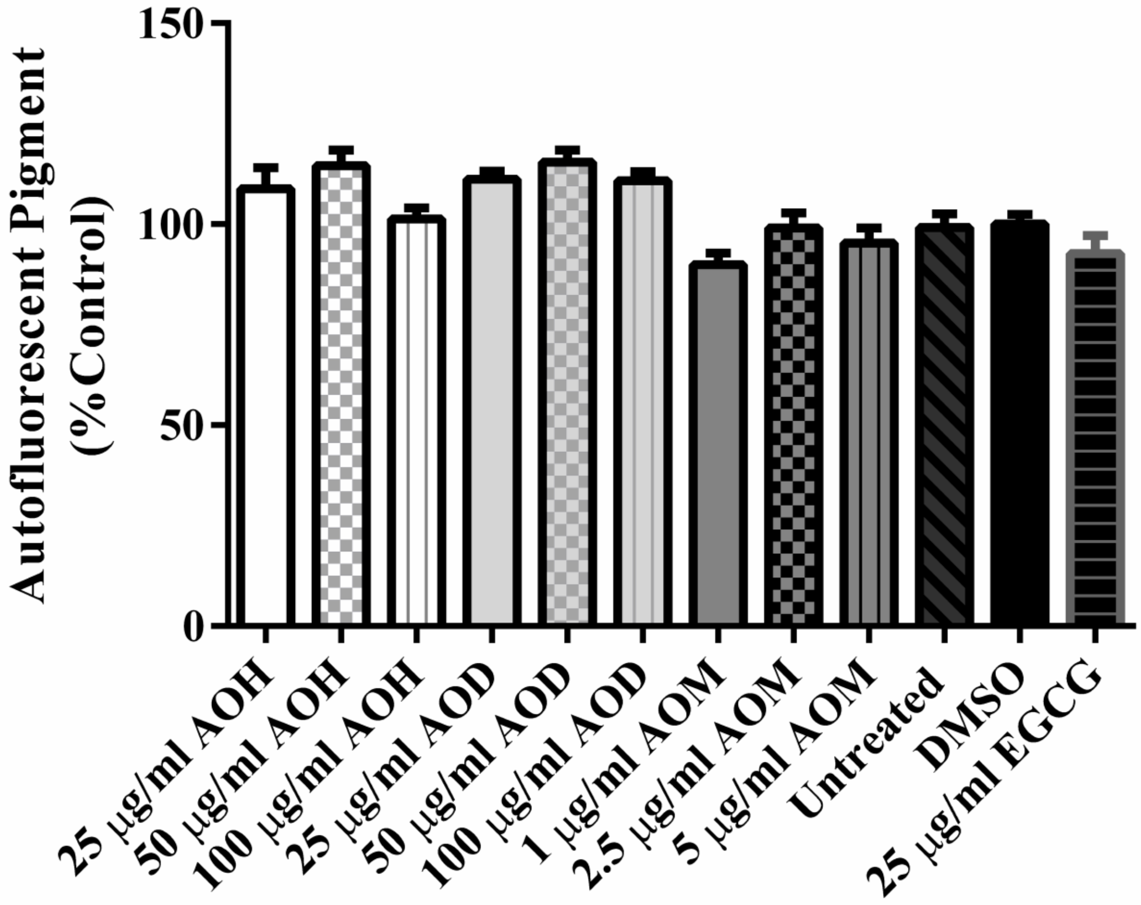

Supplementary Figure S6. AO extracts treatment had no effect on autofluorescent pigment expression. The fluorescent intensity in the worms was not affected by EGCG, DMSO and AO extracts treatment. Data are presented as the mean ± SEM (n = 100), compared to the untreated control by one-way ANOVA following Bonferroni's method (pos thoc).

Supplementary Table.4 Results and statistical analyses of the AO extracts treated *C. elegans* in lifespan assay

Strain	Treatment	Mean life span (day) ± SE	Maximum	Percentage of increased	P value	P value summary	Number of worms	Number of censored
			lifespan (days)	lifespan (vs control)	(vs control)			
N2	DMSO control	14.28 ± 0.36					N=120	N=8
N2	25 mg/ml AOH	14.91 ± 0.31	26	4.41	0.13	ns	N=126	N=18
N2	50 mg/ml AOH	17.18 ± 0.44	33	20.31	< 0.0001	****	N=137	N=8
N2	100 mg/ml AOH	15.04 ± 0.44	26	5.32	0.27	ns	N=126	N=2
N2	1 mg/ml AOM	14.76 ± 0.47	29	3.36	0.01	**	N=107	N=22
N2	2.5 mg/ml AOM	14.63 ± 0.43	29	2.45	0.27	ns	N=104	N=9
N2	5 mg/ml AOM	15.16 ± 0.49	34	6.16	0.21	ns	N=111	N=9
TK22	DMSO control	9.67 ± 0.41					N=33	N=4
TK22	25 mg/ml AOH	9.58 ± 0.30	15	-0.91	0.26	ns	N=38	N=2
TK22	50 mg/ml AOH	9.97± 0.47	16	3.16	0.85	ns	N=36	N=2
TK22	100 mg/ml AOH	10.76 ± 0.60	17	11.31	0.12	ns	N=25	N=4
TK22	1 mg/ml AOM	9.96 ± 0.46	17	3.02	0.62	ns	N=49	N=4
TK22	2.5 mg/ml AOM	10.97 ± 0.57	18	13.48	0.12	ns	N=38	N=1
TK22	5 mg/ml AOM	9.79 ± 0.56	17	1.23	0.56	ns	N=42	N=4

N2;Wild type , TK22 ;mev-1(kn1)

The life span assay was carried out with wild type (N2) and mev-1(kn-1) worms at 20 °C P-value log rank as compared with control worms; mean life span in days is the average number of days the worms survived in each group. Each treatment was compared with the control by the non-parametric log rank (Mantel−Cox) test.
